# Targeting ALK Rearrangements in NSCLC: Current State of the Art

**DOI:** 10.3389/fonc.2022.863461

**Published:** 2022-04-06

**Authors:** Ling Peng, Liping Zhu, Yilan Sun, Justin Stebbing, Giovanni Selvaggi, Yongchang Zhang, Zhentao Yu

**Affiliations:** ^1^ Cancer Center, Department of Pulmonary and Critical Care Medicine, Zhejiang Provincial People's Hospital, Affiliated People's Hospital, Hangzhou Medical College, Hangzhou, China; ^2^ Department of Medical Oncology, Shouguang Hospital of Traditional Chinese Medicine, Shouguang, China; ^3^ Division of Cancer, Department of Surgery and Cancer, Imperial College London, London, United Kingdom; ^4^ Xcovery Holdings, Palm Beach Gardens, FL, United States; ^5^ Department of Medical Oncology, Lung Cancer and Gastrointestinal Unit, Hunan Cancer Hospital, The Affiliated Cancer Hospital of Xiangya School of Medicine, Changsha, China; ^6^ Department of Thoracic Surgery, National Cancer Center, National Clinical Research Center for Cancer, Cancer Hospital and Shenzhen Hospital, Chinese Academy of Medical Sciences and Peking Union Medical College, Shenzhen, China

**Keywords:** lung cancer, ALK, rearrangement, tyrosine kinase inhibitor, resistance

## Abstract

Anaplastic lymphoma kinase (ALK) alterations in non-small cell lung cancer (NSCLC) can be effectively treated with a variety of ALK-targeted drugs. After the approval of the first-generation ALK inhibitor crizotinib which achieved better results in prolonging the progression-free survival (PFS) compared with chemotherapy, a number of next-generation ALK inhibitors have been developed including ceritinib, alectinib, brigatinib, and ensartinib. Recently, a potent, third-generation ALK inhibitor, lorlatinib, has been approved by the Food and Drug Administration (FDA) for the first-line treatment of ALK-positive (ALK+) NSCLC. These drugs have manageable toxicity profiles. Responses to ALK inhibitors are however often not durable, and acquired resistance can occur as on-target or off-target alterations. Studies are underway to explore the mechanisms of resistance and optimal treatment options beyond progression. Efforts have also been undertaken to develop further generations of ALK inhibitors. This review will summarize the current situation of targeting the ALK signaling pathway.

## 1 Background

### 1.1 ALK Signaling Pathway

NSCLC accounts for around 80% of lung cancers, with ALK+ NSCLC accounting for 3%–7% of these (1). ALK is a proto-oncogene which encodes anaplastic lymphoma kinase that is primarily expressed in the nervous system. ALK signaling is activated in cancer cells primarily through three mechanisms: gene fusions, gene amplification, and activating point mutations (2). ALK rearrangements were first identified in 2007 in NSCLC, where the 3′ region of the ALK gene was fused with the 5′ sequence of the echinoderm microtubule-associated protein-like 4 (EML4) gene. The rearrangement results in the expression of the EML4-ALK fusion protein (3). Many kinds of ALK fusion genes have been found in multiple cancer types (4). In ALK fusions, the partner drives ALK activity at the level of gene expression and through multimerization of the ALK kinase domain, which is presumed to promote several biological functions including cell differentiation, proliferation, and anti-apoptosis (5). ALK can activate signaling cascades, such as the mitogen-activated protein kinase (MAPK), (phosphatidylinositol 3−kinase) PI3K/(protein kinase B) AKT, MEK/ERK kinase 2/3 (MEKK2/3), Crk-like/CRK SH3 domain-binding guanine nucleotide-releasing factor (CRKL/C3G), Janus kinase/signal transducer and activator of transcription (JAK/STAT), and mitogen-activated protein kinase kinase 5-extracellular signal-regulated kinase 5 (MEK5-ERK5) pathways (6).

### 1.2 Diagnosis of ALK Rearrangement

The ALK locus is prone to translocation, and more than 20 different ALK fusion protein partners have been discovered (5). The detection of ALK rearrangements is widely recognized in NSCLC. Different methods are now available, with immunohistochemistry (IHC) and fluorescence *in situ* hybridization (FISH) representing validated diagnostic techniques for the assessment of ALK status (7, 8). As chromogenic *in situ* hybridization (CISH) allows concurrent analysis of histological features and gene rearrangement of the tumors, it is also a useful method in assessing ALK status (9). Next-generation sequencing (NGS) can detect a fusion between any partners, which makes it advantageous. Multiplexed PCR amplicon-based targeted NGS interrogates fusion transcripts involving many known driver genes and partners (10). Furthermore, NGS is able to assess multiple other genes simultaneously with great sensitivity.

Other than identification of ALK rearrangements from tissue biopsy, non-invasive genotyping of circulating tumor nucleic acids has gained attention as an alternative strategy. Compared to mutations and insertions/deletions, ALK rearrangements are more complex as they incorporate diverse breakpoints and multiple fusion partners (11). As DNA shedding in plasma of patients with advanced disease increases, the sensitivity of ALK fusion detection in ctDNA improves at disease progression (12, 13). The longitudinal ctDNA assays for early detection of disease progression in ALK+ patients receiving treatment is under intense investigation.

### 1.3 Characteristics of ALK+ NSCLC Patients

ALK+ NSCLC patients tend to be younger, with no smoking history, and have adenocarcinoma as the most common histological subtype (14). A recent meta-analysis confirmed that there is an increased incidence of thromboembolism in ALK+ NSCLC patients as compared to non-ALK+ patients (15). Real-world data also suggested an increased risk of venous thromboembolism in ALK-rearranged NSCLC patients (16, 17).

Advanced ALK+ NSCLC has different imaging features of primary tumor and metastatic patterns from those of EGFR+ or wild-type NSCLC (18). ALK+ NSCLC often presents with central tumor location, large pleural effusion, and absence of a pleural tail (19). ALK+ tumors are also prone to nodal metastasis and lymphangitic carcinomatosis. The radiological features can clinically help discriminate ALK+ from ALK- tumors, but genetic evidence is always required.

### 1.4 ALK Variants and Fusion Partners

ALK variants have been reported to influence the efficacy of ALK TKIs, but results were inconsistent. A prospective study from Camidge et al. did not find that different ALK variants would impact PFS for first-line alectinib or crizotinib (20). In two other studies, ALK V3a/b had a worse OS (21, 22). A recent study also suggested a prognostic role of ALK variants on treatment outcome (23). In that study, 64 ALK variants were identified in 59 patients, with V1 (32.8%) and V3a/b (28.1%) being the most common. Patients with non-V3a/b showed a trend toward longer OS. Meanwhile, although ALK+ NSCLC patients have a high PD-L1 expression rate, there is no significant association with ALK variant subtypes (23). A meta-analysis suggested that there was no significant difference of patients with the V1 variant from non-V1 in terms of PFS and OS, while V3 was associated with shorter OS (24). However, a propensity score analysis did not find a difference of ALK variants regarding clinical features and outcomes (25), which was consistent with sensitivity of ALK variants to alectinib in ALK-transformed cells (26). The molecular link between ALK variants, the differential response to TKIs, and resistance mutations support NGS-based detection of ALK status to guide treatment strategies (27).

Other than ALK variants, other ALK fusion partners include ATIC-ALK, RANBP2-ALK, NPM1-ALK, TFG-ALK, KIF5B-ALK, SQSTM1-ALK, TPM4-ALK, and CLTC-ALK (28). Their responses to ALK TKIs have been reported in several case reports, some of which were associated with better prognosis (29).

The impact of 5′-ALK on the efficacy of crizotinib was reported (30). Compared with 3′-ALK fusion alone, patients with non-reciprocal/reciprocal ALK translocation had a higher incidence of central nervous system (CNS) metastasis at baseline. Harboring non-reciprocal/reciprocal ALK translocation was an independent predictor of worse PFS for crizotinib-treated ALK\+ NSCLC.

### 1.5 Treatment Modality

As ALK+ NSCLC is a gene fusion-driven cancer, tyrosine kinase inhibitors (TKIs) have been developed to treat this unique disease. Currently, six ALK-target agents have been approved to treat advanced ALK+ NSCLC, including crizotinib, alectinib, ceritinib, ensartinib, brigatinib, and lorlatinib. These targeted agents induce durable responses and improve survival outcomes. Treatment with ALK inhibitors is recognized as the standard of care for advanced ALK+ NSCLC.

## 2 ALK Targeted Therapies in NSCLC

### 2.1 First-Generation ALK TKI

**Figure 1:**
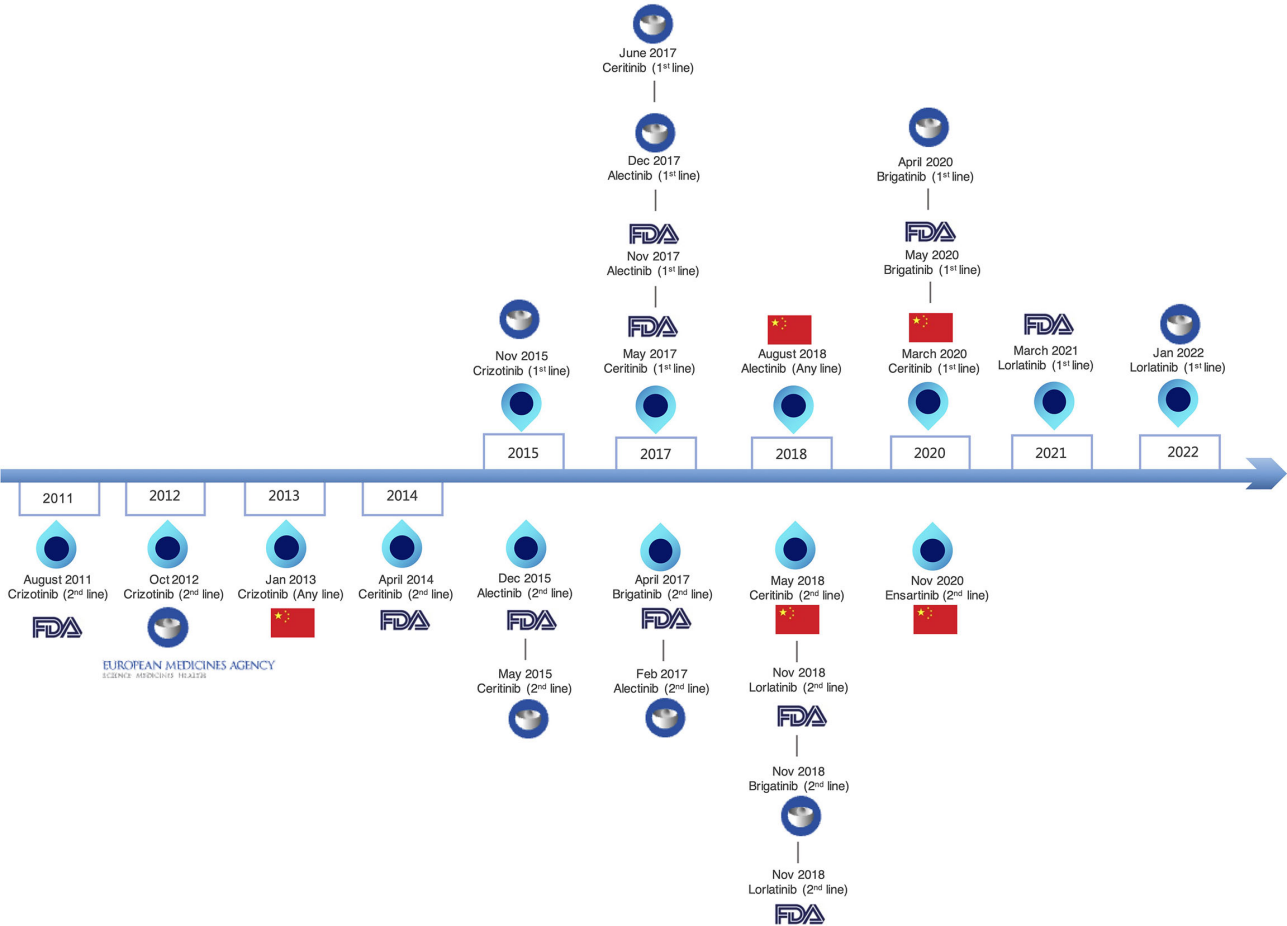
Timeline of approved ALK TKIs.

The six currently approved ALK TKIs for advanced ALK+ NSCLC were classified into three generations (**Figure 1**). The drug targets, approved indication by FDA, trial design, and primary endpoint of clinical trials are summarized in **Table 1**, which can help illustrate the currently available ALK-TKIs. The development of crizotinib, a first-in-class and first-generation ALK TKI, revolutionized the treatment of ALK+ NSCLC (52). Crizotinib is a small-molecule inhibitor of the receptor tyrosine kinases ALK, ROS1, and c-MET. In phase I and II studies, crizotinib demonstrated durable responses in advanced ALK-positive NSCLC patients (53, 54), leading to the accelerated FDA approval in 2016. In a phase III study PROFILE 1007, crizotinib showed improved PFS compared with chemotherapy in first-line and previously treated patients (34). However, the pharmacokinetic failure to crizotinib is mainly due to its poor blood–brain barrier penetration, and CNS is a common site of progression with crizotinib (55). Crizotinib-treated patients will virtually develop acquired resistance. L1196M, and G1269A, and C1156Y mutations alter the structure of the ATP-binding pocket and thus prevent crizotinib from binding to ALK (56).

**Table 1:** Pivotal clinical trials of currently approved ALK TKIs in NSCLC.

Drug	Targets	FDA approval	Study	Phase	Trial design	Region	Primary endpoint
Crizotinib	ALK, MET, ROS1 (31)	First line (January 2013)	PROFILE 1014 (32)	3	RCT	Global	PFS
			PROFILE 1029 (33)	3	RCT	Asia	PFS
		Later line (August 2011)	PROFILE 1007 (34)	3	RCT	Global	PFS
Alectinib	ALK, GAK, LTK (35)	First line (November 2017)	ALEX (36)	3	RCT	Global	PFS
			J-ALEX (37)	3	RCT	Japan	PFS
			ALESIA (38)	3	RCT	Asia	PFS
		Later line (June 2013)	ALUR (39)	3	RCT	Global	PFS
Brigatinib	ALK, EGFR, IGFR1 (40)	First line (May 2020)	ALTA-1L (41)	3	RCT	Global	PFS
		Later line (April 2017)	ALTA (42)	2	RCT	Global	ORR
Ceritinib	ALK, IGFR1, InsR, STK22D (43)	First line (May 2017)	ASCEND-4 (44)	3	RCT	Global	PFS
		Later line (April 2014)	ASCEND-5 (45)	3	RCT	Global	PFS
Ensartinib	ALK, ROS1, TRK1/2/3 (46)	First line*^a^*	eXalt3 (47)	3	RCT	Global	PFS
		Later line*^a^* (NMPA 2020)	NCT03215693 (48)	2	Single-arm	China	ORR
Lorlatinib	ALK, ROS1 (49)	First line (March 2021)	CROWN (50)	3	RCT	Global	PFS
		Later line (November 2018)	NCT01970865 (51)	2	Single-arm	Global	ORR, iORR

RCT, randomized clinical trial; TKI, tyrosine kinase inhibitor; PFS, progression-free survival; ORR, objective response rate; iORR, intracranial objective response rate; FDA, Food and Drug Administration; NMPA, National Medical Products Administration.

^a^Not approved by the FDA.

### 2.2 Second-Generation ALK TKIs

The second-generation ALK-TKIs alectinib, ceritinib, ensartinib, and brigatinib were developed to overcome crizotinib resistance, and they exhibited potent activity to crizotinib-resistant ALK+ NSCLC patients.

#### 2.2.1 Alectinib

Alectinib is a next-generation inhibitor that is highly selective for ALK (57). Alectinib, which is not a P-glycoprotein substrate, has a better penetration to the blood–brain barrier compared with crizotinib (58). Alectinib was approved by the FDA for second-line treatment in 2015 based on two single-arm trials (NP28761 and NP28673) including 225 patients treated with alectinib 600 mg orally twice daily (59). The J-ALEX trial was the first study to show that the second-generation ALK inhibitor alectinib provides a PFS advantage and is more tolerable than crizotinib with the dose of 300 mg twice daily (37). Alectinib was approved by the FDA for the first-line treatment of ALK+ NSCLC in 2017 based on the phase III ALEX trial with alectinib 600 mg twice daily (36). In a final analysis of the J-ALEX study, compared to crizotinib, alectinib did not achieve overall survival (OS) benefit (60), reflecting that the crossover to the post first-line treatment might greatly influence OS, especially in ALK+ NSCLC who could get significant benefit from all ALK TKIs. A prospective real-world study investigated the strategy of switching to alectinib in ALK+ NSCLC patients that did not experience disease progression with initial crizotinib (61). The results indicated that an early switch from crizotinib to alectinib might be a viable option and may promote better treatment compliance.

Data from J-ALEX suggested that compared with ALEX wherein 600 mg twice daily was used, alectinib 300 mg twice daily did not produce a markedly different primary outcome of PFS in a Japanese population. Since alectinib 300 mg twice daily will produce fewer adverse events (AEs) and fewer treatment interruptions, the lower dose is therefore an attractive approach in the study population (62). The on-target resistance of the mechanism of alectinib is related with emergence of G1202R and I1171N/S/T mutations (63).

#### 2.2.2 Brigatinib

In preclinical models, brigatinib (AP26113) has been shown to overcome resistance to first- and second-generation ALK TKIs (40). In crizotinib-treated (ALTA trial) and crizotinib-naïve (ALTA-1L trial) patients with ALK+ NSCLC, brigatinib has shown promising antitumor activity, including substantial activity against central nervous system (CNS) metastases (41, 64). In the final analysis of ALTA-1L, brigatinib demonstrated superior efficacy over crizotinib regardless of ALK fusion variant or TP53 mutation status, especially in patients with baseline brain metastases (65). In a network meta-analysis, brigatinib ranked the highest by efficacy in the CNS metastasis subgroup compared with alectinib, while alectinib ranked the highest by efficacy in the overall population (66). In general, brigatinib is well tolerated; however, the early-onset pulmonary toxicity has raised some concerns. The ATOMIC ARI-AT-002 trial (NCT02706626) is ongoing to evaluate the efficacy of brigatinib against ALK-resistant mutations after second-generation ALK inhibitor treatment other than brigatinib in patients with ALK+ NSCLC (67). A phase III ALTA-3 trial (NCT03596866) comparing brigatinib versus alectinib in the first-line ALK+ NSCLC is also ongoing (68).

#### 2.2.3 Ceritinib

Ceritinib obtained FDA approval for the treatment of ALK-positive patients who progressed or were intolerant to crizotinib in 2014, and as a first-line therapy in 2017. Approval was based on the ASCEND-1 (69) and ASCEND-2 studies (70). In the phase II ASCEND-2 study, crizotinib-pretreated ALK+ NSCLC received ceritinib at a standard dose of 750 mg daily and achieved an objective response rate (ORR) of 38.6% (70). A phase I, three-arm ASCEND-8 study demonstrated that ceritinib 450 mg with food showed similar efficacy and less gastrointestinal toxicity compared to 750-mg fasted (71). Two randomized Phase III trials compared ceritinib vs. standard chemotherapy in the first-line (ASCEND-4) (44) or second-line (ASCEND-5) setting (45). However, the toxicity profile of ceritinib from ASCEND-4 and ASCEND-5 indicated a higher frequency of dose interruptions and modifications due to adverse events (AEs) compared to chemotherapy. Real-world data comparing ceritinib versus alectinib in ALK+NSCLC found that alectinib exposure was associated with longer OS compared with ceritinib in ALK+ NSCLC (72). The pharmacokinetic (PK) data from the ASCEND-8 study (71) led to the FDA approval of ceritinib 450 mg QD, administered with food.

#### 2.2.4 Ensartinib

Ensartinib (X-396) is an aminopyridazine-based small molecule that inhibits ALK. Furthermore, ensartinib has reported some activity against ROS1, AXL, and cMET (73). In a phase 1/2 trial, ensartinib has shown promising clinical activity in ALK+ NSCLC (46). A single-arm phase 2 trial investigating ensartinib in second-line ALK+ NSCLC demonstrated an ORR of 52% (48), which led to its approval by the National Medical Products Administration (NMPA) of China. The phase III eXalt3 study comparing ensartinib versus crizotinib for the first-line treatment of ALK+ NSCLC demonstrated that ensartinib is superior to crizotinib in both systemic and intracranial diseases (47). Of note, crossover was not allowed in this trial. A dynamic sequencing of circulating tumor DNA (ctDNA) in ensartinib-resistant ALK+ NSCLC patients revealed that ALK-dependent resistance mechanisms of ensartinib were mainly due to G1269A, G1202R, and E1210K mutations (74).

### 2.3 Third-Generation ALK TKI

Approximately half of resistance to second-generation ALK-TKIs is associated with secondary mutations in the ALK kinase domain (75). Lorlatinib is a 3^rdz^-generation ALK TKI and is a small and compact macrocyclic inhibitor. The macrocyclic formation had an improved metabolic stability and a low frequency of P-glycoprotein-mediated efflux *in vitro*. Diverse compound ALK mutations were identified in lorlatinib-resistant cells or patient samples after sequential ALK-TKI treatments (76, 77). Lorlatinib can inhibit G1202R mutation, but not compound mutations (78). Lorlatinib was approved by the FDA in 2018 for the second- or third-line treatment of ALK+ NSCLC (51). The phase III CROWN study comparing lorlatinib versus crizotinib achieved the best-in-class differential PFS benefit of HR 0.28 (50), which led to its first-line approval of the FDA in March 2021. Crossover was not allowed in the CROWN study. This result may redefine the new potential standard of care in the first-line setting. As there are no head-to-head comparisons of lorlatinib to second-generation ALK TKIs, debates were raised regarding whether lorlatinib is the best first-line treatment for ALK+ NSCLC (79, 80). Compared with alectinib, lorlatinib was associated with a higher incidence of grade 3 or higher AEs (81) mostly related to its higher penetration in the CNS.

### 2.4 Fourth-Generation ALK TKIs Under Investigation

The sequential use of ALK TKIs which is active to ALK “single mutant” will lead to double ALK resistance mutations. Fourth-generation ALK TKIs such as TPX-0131 and NVL-655 have been developed, which are “double mutant active.” TPX-0131 is a compact macrocyclic inhibitor, which was designed to fit completely in the ATP-binding pocket. It may reduce the susceptibility to a variety of ALK TKI-resistant mutations, including solvent front, hinge region, gatekeeper, and compound mutations (82). Other than being sensitive to most single resistant mutations, TPX-0131 is effective for compound mutations such as G1202R+L1198F, G1202R+L1196M, L1196M+ L1198F, and G1202R+C1156F. Another 4th-generation ALK TKI, NVL-655, is a brain-penetrant small-molecule inhibitor with activity against solvent front drug-resistance mutations, such as G1202R, G1202R+L1196M, and G1202R+G1269A (83). Furthermore, NVL-655 displayed brain penetrance to open up the potential to treat brain metastases while avoiding off-target CNS adverse events.

### 2.5 Other ALK TKIs

Entrectinib is a selective inhibitor of TRKA/B/C, ALK, and ROS1 (84). Combined results from two phase I/II basket trials (ALKA-372-001 and the STARTRK-1 trial) suggested that entrectinib was well tolerated and active against ALK+ NSCLC (85). A phase II basket trial STARTRK-2 (NCT02568267) is currently ongoing to evaluate entrectinib for the treatment of patients with NTRK, ROS1, and ALK gene rearrangements. Repotrectinib (TPX-0005) is a rationally designed macrocyclic TKI developed to inhibit ALK, ROS-1, and TRKA-C (86). It is smaller than lorlatinib and has a high activity in CNS. The TREDENT-1 study (NCT03093116) for repotrectinib showed encouraging data in ALK+ NSCLC patients (87).

Other novel ALK TKIs include TQ-B3139 (88), WX-0593 (89), PLB-1003 (90), SAF-189s (91), and CT-707 (92). Several other ALK TKIs are under preclinical investigation, such as gilteritinib (93) and XMU-MP-5 (94). An ALK proteolysis-targeting chimeric (PROTAC) degrader is also under development. The six different ALK PROTACs are all based on the second-generation ALK-TKIs, including ceritinib-based (95–98), TAE684-based (96), and brigatinib-based ALK PROTACs (99). During this process, kinase mutations and off-target effects may occur, which is a major clinical challenge (100). The ongoing clinical trials investigating novel-generation ALK TKIs in ALK+ NSCLC are summarized in **Table 2** (up to December 18, 2021).

**Table 2:** Ongoing clinical trials of novel ALK TKIs against ALK-arranged NSCLC.

Clinical trial identifier	Study design	Intervention	Setting	Primary endpoint	Phase	Status
NCT04009317	260 participants Parallel assignment Randomized, open label	TQ-B3139 vs. crizotinib	First line	PFS	3	Recruiting
NCT04632758	330 participants Parallel assignment Randomized, open label	WX-0593 vs. crizotinib	First line	PFS	3	Recruiting
NCT04056572	135 participants Single-group assignment Non-randomized, open label	TQ-B3139	Second line	ORR	2	Recruiting
NCT04641754	176 participants Single-group assignment Non-randomized, open label	WX-0593	Second line	ORR	2	Recruiting
NCT04211922	104 participants Single-group assignment Non-randomized, open label	Alkotinib	Second line	ORR	2	Recruiting
NCT02568267	60 participants (basket) Single-group assignment Non-randomized, open label	Entrectinib (RXDX-101)	Second line	ORR	2	Recruiting
NCT03093116	500 participants (basket) Single-group assignment Non-randomized, open label	Repotrectinib (TPX-0005)	Second line	DLT, RP2D, ORR	1/2	Recruiting
NCT04849273	210 participants Single-group assignment Non-randomized, open label	TPX-0131	Second line	DLT, RP2D, ORR	1/2	Recruiting
NCT04237805	280 participants Single-group assignment Non-randomized, open label	SAF-189s (foritinib)	First/second line	DLT, ORR	1/2	Recruiting
NCT03130881	60 participants Single-group assignment Non-randomized, open label	PLB1003	Second line	DLT	1	Recruiting
NCT03607188	18 participants Single-group assignment Non-randomized, open label	Alkotinib	Second line	DLT	1	Recruiting
NCT05055232	120 participants Single-group assignment Non-randomized, open label	XZP-3621	Second line	Toxicity, DLT, MTD	1	Recruiting
NCT02695550	40 participants Single-group assignment Non-randomized, open label	CT-707	Second line	DLT, toxicity	1	Unknown

PFS, progression-free survival; ORR, objective response rate; DLT, dose-limiting toxicity; RP2D, recommended phase 2 dose; MTD, maximum tolerated dose.

### 2.6 Treatment Options Other Than ALK TKIs

#### 2.6.1 Chemotherapy

As chemotherapy has limited efficacy in ALK+ NSCLC after failure of a second-generation ALK TKI, combination therapy with ALK TKI and chemotherapy has been proposed in ALK+ NSCLC refractory to at least one second-generation ALK TKI. This strategy has been proved to be a possible choice by several studies. Crizotinib plus pemetrexed in ALK+ NSCLC patients with multiple CNS metastases demonstrated better efficacy than monotherapy (101). Chemotherapy in combination with ALK TKI proved to be of higher efficacy, suggesting a potential role for ongoing ALK inhibition (102).

#### 2.6.2 Anti-Angiogenic Drugs

Anti-angiogenic drugs have also been investigated in ALK+ NSCLC. Vascular endothelial growth factor (VEGFR) expression has been reported to be upregulated in ALK+ NSCLC, which induces resistance to ALK TKIs (103). A single-arm study of involving 12 patients of ALK+ NSCLC demonstrated that crizotinib plus bevacizumab showed benefit in first-line ALK+ NSCLC, with an acceptable safety profile (104). In another phase 1/2 single-arm trial, alectinib plus bevacizumab was also well tolerated (105).

#### 2.6.3 Immune Checkpoint Inhibitors

The PD-L1-positive and strongly positive rates among ALK+ NSCLC patients were 46.7%–50% and 13.3%–16%, respectively (23, 106). Studies have shown that the ALK oncoprotein is able to upregulate PD-L1 expression in lung cancer cells. Upregulation of PD-L1 by EML4-ALK was mediated by activating MEK-ERK and PI3K-AKT signaling pathways in NSCLC, which suggests a link between oncogene and PD-L1 expression (107). The expression of PD-L1 in ALK+ NSCLC has brought immunotherapy drugs such as immune checkpoint inhibitors (ICIs) into consideration for ALK+ NSCLC. A real-world analysis of ICIs in ALK+ NSCLC patients from a Flatiron Health electronic health record demonstrated limited efficacy of ICIs provided either before or after TKIs (108). Recent evidence indicated new roles of ALK and its genetic aberrations in immune evasion and in innate and cell-mediated immunity (109). The tumor microenvironment of ALK+ NSCLC suggested a poorly immunogenic “immune desert” of ALK+ NSCLC that also prevents the successful use of immune checkpoint inhibitors (ICI) (110). Furthermore, the toxicity of ICI for ALK+ NSCLC patients was too high. The sequential use of ICIs and crizotinib has also been reported with an increased risk of hepatotoxicity in retrospective studies (111). The challenge to researchers is not only to improve the efficacy of ICI in ALK+ NSCLC but also to find immunotherapeutic drugs that have acceptable toxicity in combination regimens.

#### 2.6.4 Radiotherapy

There are no firm data for concurrent usage of ALK TKIs and radiotherapy. However, radiotherapy acts as a salvage treatment for patients who have oligoprogressive metastatic disease while under targeted therapy (112). In oligoprogressive diseases of ALK+ lung cancer, continuation of ALK TKIs with local ablative therapy should be considered for sustained control, which can potentially eradicate resistant cancer cell clones and confer survival benefit (113). Ablative and hypofractionated radiotherapy is one strategy for ALK+ lung cancer, since many ALK+ NSCLC patients treated with ALK TKIs experienced local disease progression (114). Timing of radiotherapy remains unclear, especially under different clinical settings. Furthermore, the safety of the combination of ALK TKIs and radiotherapy is unclear (115). Case reports using radiotherapy combined with alectinib and lorlatinib presented radiation-induced CNS necrosis, and this toxicity remains long after radiation (116, 117).

## 3 Discussion

### 3.1 How to Choose the Optimal First-Line Treatment?

**Figure 2:**
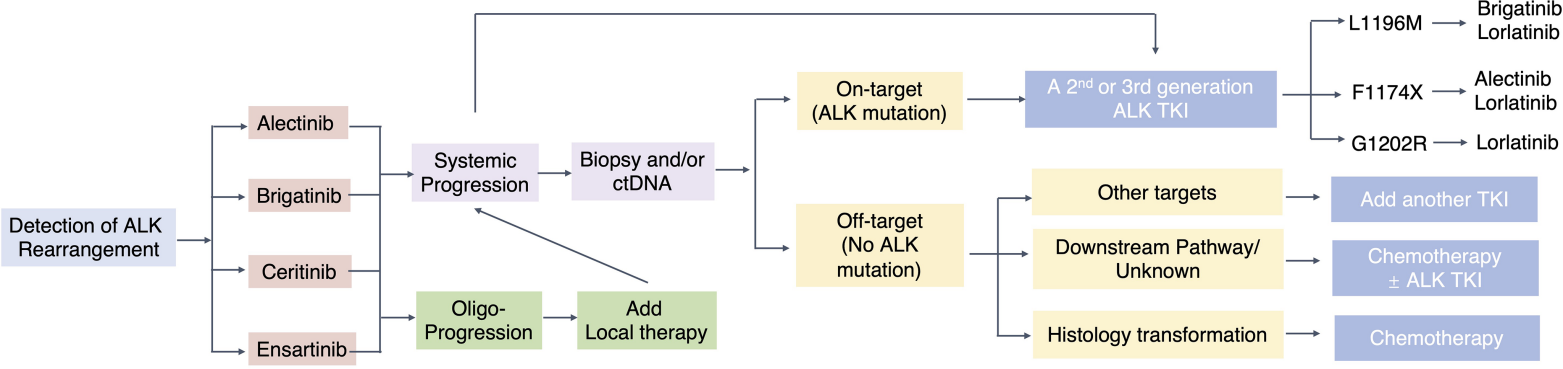
Treatment strategy for ALK+ NSCLC.

There is a continuous debate regarding the choice of the optimal upfront ALK TKI for the first-line treatment of ALK+ NSCLC, the subsequent sequencing strategies, and whether these considerations should be based on specific on-target ALK resistance mutations or not. Our recently published Bayesian network meta-analysis has compared the efficacy and safety of 6 ALK TKIs and chemotherapy in the first-line setting (118). Regarding PFS benefit for the first-line setting, lorlatinib ranks first, while the toxicity of lorlatinib needs to be paid attention to. However, the goal of treating advanced ALK+ NSCLC should not just be limited to improve median PFS in the first-line setting. There is no consensus on how to best sequence the ALK TKIs which are “single mutant active.” Some advocate using second-generation ALK TKIs due to their favorable toxicity profile, while leaving lorlatinib, the only third-generation ALK TKI, for salvage treatment (**Figure 2**).

ALK+ NSCLC has a high tendency for brain metastases compared to non-oncogene-driven NSCLC subtypes (119). Compared with first-generation ALK TKI, second- and third-generation ALK TKIs have a better efficacy of brain metastases. Ceritinib demonstrated an intracranial ORR of 35%–73% and an intracranial disease control rate (DCR) of 61%–86% in ALK-TKI naïve and -pretreated patients (44, 45, 69, 70). The intracranial ORR and intracranial DCR of alectinib in clinical trials were 54%–81% and 78%–90%, respectively (36, 39, 120, 121). Brigatinib showed an encouraging activity in the CNS, with an intracranial ORR of 42%–73% and an intracranial DCR of 83%–93% (42). A meta-analysis investigated the role of ALK TKIs in the treatment of ALK+ NSCLC patients with brain metastases, who had been pretreated with radiotherapy or not and/or chemotherapy (122). The results also confirmed better intracranial control with second-generation ALK TKIs (alectinib, brigatinib, and ceritinib) compared with crizotinib. Ensartinib demonstrated an intracranial ORR of 63.6%–70% and an intracranial DCR of 98%–100% (47, 48). Lorlatinib had an intracranial ORR of 61%–66% in the first-line setting (50). Lorlatinib also showed substantial intracranial activity in second-generation ALK TKI-pretreated patients, with or without baseline CNS metastases (123, 124). This evidence suggested that withholding brain radiotherapy in patients with asymptomatic brain metastases and use of radiotherapy during progression could be an option. Prospective trials are warranted to confirm the validity of this strategy.

### 3.2 Resistance Mechanism of ALK TKIs

There are two main categories of resistance mechanisms to ALK TKIs, namely, on-target alterations such as ALK mutation/gene amplification and off-target changes such as bypass signaling pathways (75). Substitution with ALK-destabilizing mutations could activate the ALK signaling pathway, which confers drug resistance to inhibitors (125). Inherent ALK resistance mutations are only found in a proportion of patients with acquired resistance to ALK-TKI, for first- and second-generation ALK-TKIs. ALK mutations such as somatic kinase domain mutations are the primary resistant mechanism. Two major ALK mutations after first-generation ALK TKI crizotinib were L1196M (7%) and G1269A (4%) (75), which alter 3D conformation and hinder TKI binding (126). Resistance to second-generation ALK TKIs is associated with specific mutations, such as G1202R, I1171N, S1206Y, and E1201K, for which not all TKIs are equally effective. In patient samples post-ceritinib, secondary mutations were detected in 56% of the cases, with 17% of double mutations: G1202R (21%), F1174 C/L (17%), and C1156Y (8%) (75). Acquired mutations of alectinib have been identified in 53% of the patients: G1202R (29%), I1171T/S (12%), V11180L (6%), and L1196M (6%) (75). Although brigatinib showed activity against G1202R, which is a frequent mutation associated with alectinib-resistant cancer (127), it is worth noting that G1202R has also been detected in brigatinib-resistant samples, raising the question of how clinically useful brigatinib is against this solvent front mutation (128). Of note, G1202R was not the most common ALK mutation in ensartinib-resistant patients, in which G1269A (6.6%) was the more identified than G1202R (2.8%) among 14.2% of the patients with secondary ALK mutations post second-line ensartinib (74). On-target resistance to the third-generation ALK inhibitor lorlatinib is primarily mediated by compound ALK mutations (129). Interestingly, some compound mutations that lead to lorlatinib resistance result in re-sensitization to first- or second-generation ALK TKIs, such as I1171N + L1256F, and C1156Y + L1198F which lead to re-sensitization to alectinib and crizotinib, respectively (76, 130). Patients with secondary ALK mutations refractory to the previous ALK TKI can be treated with other ALK TKIs. This re-sensitization phenomenon supported the sequential and possibly alternating use of different ALK TKIs.

ALK-independent mechanisms are only partially understood and particularly challenging, as they may result in refractoriness to further ALK inhibition. ALK-independent resistance mechanisms involve bypass pathways, such as EGFR, cMET, and AXL, or histological transformation into small cell lung cancer (SCLC) (131–133). Mechanisms of resistance to novel generation ALK TKIs are complex and diverse, reflecting the selective genetic pressure of drugs (134). In a prospective MATCH-R study, adaptive mechanisms driving resistance to lorlatinib were explored by a longitudinal assessment of tumor biopsies and ctDNA and the development of patient-derived xenograft (PDX) and cell lines (135). Epithelial–mesenchymal transition (EMT) mediated resistance in two patient-derived cell lines, and a novel bypass mechanism of resistance caused by NF2 loss-of-function mutations was described.

### 3.3 Toxicity Considerations

Clinical trials have established that ALK TKIs are generally safe and well tolerated. First-generation crizotinib has demonstrated a spectrum of toxicities, such as visual disorders (diplopia, photopsia, blurred vision), as well as QTc prolongation and bradycardia, while most of the AEs are grades 1–2 (136). Gastrointestinal toxicities were associated with different ALK TKIs, such as vomiting, nausea, and diarrhea. Brigatinib was characterized by a peculiar and early-onset interstitial lung toxicity (137). The most common AEs of lorlatinib were notably hypercholesterolemia (81%) and hypertriglyceridemia (60%), with cases of grade 3–4 toxicities occurring in 16% of patients. Special AEs of lorlatinib include CNS effects such as changes in mood, mental status, and peripheral neuropathy (138). Although different ALK TKIs share some common AEs, they have some unique toxicities, which should be taken into account to identify the right drug for the right patient. Finding ways to tackle these toxicities will play an essential role in drug strategies for ALK+ NSCLC patients.

A list of different parameters could potentially affect the interpretation of toxicity (139). Among them, the drug dose is one of the reasons which influence the tolerability and toxicity of ALK TKIs. As toxicity is related to drug dose, fewer toxicities were noted with the 300-mg dose than with the 600-mg dose of alectinib (62). Exposure-response analyses indicated that a lower dose of alectinib and crizotinib could result in diminishing treatment efficacy (140). Therefore, monitoring drug dose and toxicity might influence the treatment outcome of patients receiving ALK TKIs.

### 3.4 Beyond Advanced NSCLC

The treatment strategy of advanced ALK+ NSCLC has brought ALK-targeted therapy into early and locoregional (N2) stages. As acquired resistance of targeting ALK in the advanced stage setting emerges inevitably, TKIs are able to inhibit cancer cell proliferation, hinder tumor growth, and control cancer metastasis, but not to eradicate or cure the disease. There are no clear data regarding the frequency in early-stage or locoregional disease (141). Inhibiting the ALK signaling pathway at earlier stages still faces many challenges. Neoadjuvant and adjuvant ALK TKIs in ALK+ NSCLC have yielded mixed results (142). **Table 3** shows the clinical trials of ALK TKIs in neoadjuvant and adjuvant settings (up to December 18, 2021).

**Table 3:** Clinical trials using ALK TKIs in neoadjuvant and adjuvant settings of ALK-arranged NSCLC.

Clinical trial identifier	Study design	Intervention	Setting	Primary endpoint	Phase	Status
NCT03456076	255 participants Parallel assignment Randomized, open label	Alectinib vs. chemotherapy	Adjuvant	DFS	3	Recruiting
NCT02201992	168 participants Parallel assignment Randomized, open label	Crizotinib vs. observation	Adjuvant	OS	3	Recruiting
NCT04302025	60 participants Single group assignment Non-randomized, open label	Alectinib	Neoadjuvant	MPR	2	Recruiting
NCT05015010	33 participants Single group assignment Non-randomized, open label	Alectinib	Neoadjuvant	MPR	2	Recruiting
NCT03088930	3 participants Single group assignment Non-randomized, open label	Crizotinib	Neoadjuvant	ORR	2	Completed

DFS, disease-free survival; MPR, major pathological response; OS, overall survival; ORR, objective response rate.

## 4 Conclusions

In this “precision medicine” era, although the detection of oncogenes is common practice and the administration of targeted agents is a recognized option, molecular results should be interpreted with caution. The integration of the roles including pathologists, molecular biologists, and clinicians is needed. The treatment algorithm of ALK+ NSCLC is becoming more complex. New-generation TKIs have better CNS penetration across the blood–brain barrier, resulting in superior intracranial response rates and preventing brain metastases. A head-to-head comparison between all ALK TKIs is still lacking, but novel ALK TKIs are being developed to overcome resistance to currently available ALK TKIs, hypothesizing a defined sequential ALK TKI strategy in this disease. After failure of targeted therapies, chemotherapy might still be a valid option, while the role of immunotherapy is yet to be clarified. Overcoming the challenges for the development of more potent drugs will be essential to improving the survival rate of ALK+ NSCLC in the future.

## Author Contributions

Conceptualization, LP and YZ. Writing—original draft preparation, LP and ZY. Writing—review and editing, LP, JS, GS, and YS. Supervision, JS and ZY. All authors contributed to the article and approved the submitted version.

## Funding

This study was supported by a grant from the Administration of Traditional Chinese Medicine of Zhejiang Province (grant number: 2022ZA021) and a grant from the Medical Science Research Foundation of Health Bureau of Zhejiang Province (grant number: 2022KY545).

## Conflict of Interest

GS is employed by, and holds stock in, Xcovery Holdings, Inc. JS is Editor-in-Chief of Oncogene and has sat on SABs for Vaccitech, Heat Biologics, Eli Lilly, Alveo Technologies, Pear Bio, Agenus, Equilibre Biopharmaceuticals, Graviton Bioscience Corporation, Celltrion, Volvox, Certis, Greenmantle, vTv Therapeutics, APIM Therapeutics, Bryologyx and Benevolent AI. He has consulted with Lansdowne partners and Vitruvian. He chairs the Board of Directors for Xerion and previously BB Biotech Healthcare Trust PLC.

The remaining authors declare that the research was conducted in the absence of any commercial or financial relationships that could be construed as a potential conflict of interest.

## Publisher’s Note

All claims expressed in this article are solely those of the authors and do not necessarily represent those of their affiliated organizations, or those of the publisher, the editors and the reviewers. Any product that may be evaluated in this article, or claim that may be made by its manufacturer, is not guaranteed or endorsed by the publisher.
